# Heritability and Pedigree Analyses of Hypertrophic Cardiomyopathy in Rhesus Macaques (*Macaca Mulatta*)

**DOI:** 10.3389/fvets.2021.540493

**Published:** 2021-06-02

**Authors:** Yu Ueda, Samantha Kovacs, Rachel Reader, Jeffrey A. Roberts, Joshua A. Stern

**Affiliations:** ^1^Department of Clinical Sciences, College of Veterinary Medicine, North Carolina State University, Raleigh, NC, United States; ^2^Department of Medicine & Epidemiology, School of Veterinary Medicine, University of California, Davis, Davis, CA, United States; ^3^California National Primate Research Center, University of California, Davis, Davis, CA, United States

**Keywords:** left ventricular hypertrophy, mode of inheritance, pedigree analysis, cardiac disease, genetics

## Abstract

In a colony of rhesus macaques at California National Primate Research Center (CNPRC), naturally occurring hypertrophic cardiomyopathy (HCM) classified by left ventricular hypertrophy without obvious underlying diseases has been identified during necropsy over the last two decades. A preliminary pedigree analysis suggested a strong genetic predisposition of this disease with a founder effect. However, the mode of inheritance was undetermined due to insufficient pedigree data. Since 2015, antemortem examination using echocardiographic examination as well as other cardiovascular analyses have been performed on large numbers of rhesus macaques at the colony. Based on antemortem examination, HCM was diagnosed in additional 65 rhesus macaques. Using HCM cases diagnosed based on antemortem and postmortem examinations, the heritability (h^2^) was estimated to determine the degree of genetic and environmental contributions to the development of HCM in rhesus macaques at the CNPRC. The calculated mean and median heritability (h^2^) of HCM in this colony of rhesus macaques were 0.5 and 0.51 (95% confidence interval; 0.14–0.82), respectively. This suggests genetics influence development of HCM in the colony of rhesus macaques. However, post-translational modifications and environmental factors are also likely to contribute the variability of phenotypic expression. Based on the pedigree analysis, an autosomal recessive trait was suspected, but an autosomal dominant mode of inheritance with incomplete penetrance was also possible. Further investigation with more data from siblings, offspring, and parents of HCM-affected rhesus macaques are warranted. Importantly, the findings of the present study support conducting genetic investigations such as whole genome sequencing to identify the causative variants of inherited HCM in rhesus macaques.

## Introduction

Hypertrophic cardiomyopathy (HCM) is the most common inherited cardiomyopathy in humans. More than 20 genes with 1,500 genetic variants have been identified over the last couple decades (1, 2). Among these genes, mutations in the sarcomeric genes especially myosin heavy chain and myosin binding protein C genes are most commonly associated with HCM development in humans. The majority of HCM cases have been reported as a single gene disorder with an autosomal dominant pattern of inheritance (3–5). However, the phenotypic expression of HCM varies significantly with incomplete penetrance in concert with various other genetic and epigenetic influences. Identifying the causality of these genetic variants on the development of HCM is challenging due to the complexity of genotypic and phenotypic interactions (6).

In a colony of rhesus macaques at the California National Primate Research Center (CNPRC), a large number of rhesus macaques were diagnosed with HCM based on necropsy over a 22-year period (7). Diagnosis of HCM in the rhesus macaques was made based on prominent left ventricular concentric hypertrophy with reduced left ventricular chamber size similarly noted in human HCM cases (8). Among these HCM-affected rhesus macaques, approximately half were diagnosed after sudden death, which is the most common sign in association with HCM in both rhesus macaques and young human adults (7, 9, 10). Based on the similarity of gross and histological abnormalities as well as clinical presentation of HCM in rhesus macaques and HCM in humans, this disease was thought to be an excellent non-human primate model of HCM to help improve our understanding of this complex disorder. Nevertheless, left ventricular hypertrophy also results from other diseases and conditions including systemic hypertension, aortic and subaortic stenosis, infiltrative cardiomyopathies, and intense prolonged endurance. Therefore, these underlying diseases and abnormalities need to be ruled out before diagnosing inherited HCM (11). In addition, epigenetic influence needs to be considered when genotypic and phenotypic interaction of HCM is determined. This could be accomplished by performing a pedigree analysis and calculating heritability estimation of HCM in rhesus macaques (12).

A preliminary pedigree analysis performed in the same colony of rhesus macaques suggested a genetic predisposition for this disease because HCM-affected rhesus macaques were all descended from a small number of founders (13). In humans, HCM is known to be inherited with an autosomal dominant pattern with incomplete penetrance (1, 14), but the findings in the preliminary pedigree analysis of HCM in rhesus macaques diagnosed based on necropsy were not consistent with that of human HCM (13). This inconsistency could be due to a lack of phenotypic data in many offspring, siblings, and parent rhesus macaques of HCM-affected animals. Since 2015, antemortem examination of rhesus macaques with HCM have been implemented in the colony of rhesus macaques and more phenotypic data has been obtained from HCM-affected rhesus macaques (15). These HCM cases were diagnosed based on echocardiographic examination with a thickened left ventricular wall and concurrent left ventricular diastolic dysfunction. Further antemortem phenotyping including electrocardiographic, thoracic radiographic, and biomarker analyses of cardiac troponin I and N-terminal pro-brain natriuretic peptide were also shown to be used as adjunctive tests to identify rhesus macaques with HCM (16–18).

In this study, using the cumulative HCM cases and healthy controls diagnosed by postmortem and antemortem examination, the heritability of HCM in rhesus macaques at CNPRC was determined. Furthermore, pedigree analysis was performed with a large sample number of HCM cases in the colony of rhesus macaques to determine the mode of inheritance.

## Materials and Methods

### Subjects and Housing

The pedigree and heritability analyses were conducted in a colony of rhesus macaques at the CNPRC. This CNPRC is an USDA-registered and AAALAC-accredited facility and maintains an IACUC approval from the University of California-Davis and a Public Health Service Assurance.

All animals in the facility are taken care of in accordance with the Animal Welfare Act and the Guide for the Care and Use of Laboratory Animals. They are either housed in outdoor or indoor cages. Rhesus macaques in the outdoor cages are housed as groups in 0.5-acre rectangular enclosures. Rhesus macaques in the indoor cages are usually housed as pairs in stainless steel cages sized correspondingly to primary cage-space regulations. Some rhesus macaques are housed singly in the indoor cages if single housing is indicated for research or medical purpose. The indoor rhesus macaques were housed in a room with alternating 12 h: 12 h light and darkness. All rhesus macaques were housed with species-appropriate environmental enrichment. Animals were fed commercial primate chow twice daily (LabDiet Monkey Diet 5,047, Purina Mills International, St Louis, MO) with fruit and vegetable supplements provided biweekly. Water is provided using an automatic watering device to animals without any access restriction. General physical examination, tuberculosis testing, dental prophylaxis, and baseline blood tests including complete blood count analysis and serum biochemistry are performed accordingly. When any health issues are noted other than the cardiac disease of interest, the rhesus macaques were transferred to a separate colony and excluded from the present study. Surveillance for bacterial and viral infection (herpes B virus, simian T-lymphotropic virus, SIV, and simian type D retrovirus) is conducted for all rhesus macaques.

### Postmortem Gross Pathology Diagnosis of HCM

A gross necropsy of rhesus macaques was completed by veterinary pathologists after sudden death, elective euthanasia for terminal studies, or euthanasia due to poor medical conditions. Prior to 2007, HCM was diagnosed based on subjective observation of a markedly thickened left ventricular wall with apparently obliterated left ventricular lumen. After 2008, HCM diagnosis was protocolized based on the ratio of the left ventricular external diameter to internal luminal diameter (O: I ratio). The O:I ratio was obtained by sectioning the left ventricle transversely midway between the apex and the base of the heart (0.5 to 1 cm below the aortic valve) and pass through the body of the papillary muscles. The O: I ratio >3 was considered to be HCM-positive based on previously reported results (7). The pathology reports were reviewed for previously diagnosed HCM animals and cases with incomplete records were excluded from the pedigree and heritability analyses.

### Antemortem Echocardiographic Diagnosis of HCM

Rhesus macaques were sedated with ketamine hydrochloride (10 mg/kg IM, Ketaject, Phoenic Pharmaceutical, St. Joseph, MO) 5 to 10 min prior to echocardiographic examination. Echocardiographic examination of rhesus macaques was performed as previously described as a part of routine screening for other ongoing projects (15, 19). All echocardiographic examination was performed by a veterinary cardiologist (JS) or a cardiology research fellow (YU) under the direct supervision of a veterinary cardiologist. One of two echocardiographic ultrasound devices (Affiniti 50, Phillips, Best, Netherland, and CX50 Ultrasound System, Phillips, Best, Netherland) was used for echocardiographic examination with animals positioned in right and left lateral recumbency, subsequently. Echocardiographic measurements were done on three consecutive heart cycles using a leading-edge-to-leading edge technique (20). HCM was diagnosed when animals have a thickened left ventricular wall and diastolic dysfunction. When rhesus macaques possess only one of these abnormalities, they were considered equivocal cases. A thickened left ventricular wall was diagnosed when the M-mode images from right parasternal short axis view at the chordae level exceeds the following cut-off values: rhesus macaques younger than 9 years old with interventricular septal thickness during diastole or left ventricular posterior wall thickness during diastole greater than or equal to 6.5 mm and animals older than 9 years old with interventricular septal thickness during diastole greater than or equal to 8.8 mm or left ventricular wall thickness during diastole greater than or equal to 7.4 mm. The cut-off values were set based on previously published studies (15, 19). The cut-off values were <2 standard deviation from the means in healthy rhesus macaques reported in previous studies (19, 21, 22). Diastolic dysfunction was determined based the measurements of the passive early filling velocity (E wave) to atrial contraction later filling velocity (A wave) ratios measured on left apical four-chamber view spectral Doppler images, and measurement of lateral or medial mitral annulus motions and peak velocities measured in early (E' wave) to later diastole (A' wave) ratios on the color tissue doppler image on the left apical four-chamber view. Criteria for diagnosing diastolic dysfunction was a transmitral E: A ratio less than or equal to 0.9 or a lateral or medial E': A' ratio less than or equal to 0.9. Criteria for diagnosing diastolic dysfunction was a transmitral E: A ratio less than or equal to 0.9 or a lateral or medial E': A' ratio less than or equal to 0.9 (15, 19).

### Pedigree Analysis and Mode of Inheritance

Historical pedigree data were obtained from the database at the CNPRC. All rhesus macaques without a presence of HCM determined based on the necropsy or echocardiographic examination were considered as controls. Rhesus macaques with thickened left ventricular wall identified on the gross pathology with or without I: O ratio more than three, and with thickened left ventricular wall with diastolic dysfunction diagnosed on echocardiographic examination were included as HCM positive cases. The equivocal HCM cases with either a thickened left ventricular wall or diastolic dysfunction were excluded from the pedigree and heritability analyses. Rhesus macaques without any necropsy or echocardiographic data were considered as unknown phenotype status. The pedigree was drawn by hand using Adobe Illustrator. A proposed mode of inheritance was determined using previously established definition of autosomal, x-linked, recessive and dominant traits (23).

### Statistical Analysis

Heritability analysis was performed with a binary categorical analysis (i.e., HCM vs. control) using the MCMCglmm Package in R-software (24) (Supplementary Table 1). The underlying unobservable risk for developing HCM is assumed to be continuous, however the diagnosis of HCM only occurs when the risk exceeds a threshold of Tao = 0. A generalized mixed model with a probit link function was used. An estimated mean and median heritability (h^2^) with a 95% confidence interval for HCM were then calculated. Variance component analysis was not performed due to small sample sizes. The frequency of HCM identified in male and female rhesus macaques were compared using a Chi-squared test.

## Results

A total of 1,741 (745 males and 996 females) were included in the heritability and pedigree analysis. Of 1,741 rhesus macaques, 1,096 animals have unknown HCM status, 502 (256 males and 246 females) were controls and 143 (73 males and 70 females) rhesus macaques were diagnosed with HCM in this colony at the time of writing. Of these, 78 rhesus macaques (39 males and 39 females) were diagnosed with HCM during necropsy and 65 rhesus macaques (31 males and 34 females) were diagnosed with HCM by antemortem echocardiographic examination. A total of 260 rhesus macaques were diagnosed as equivocal HCM and excluded from the analyses. There was no statistically significant difference in the frequency of HCM diagnosis in males and females (*p* = 0.15). Of 143 rhesus macaques with HCM, 66 HCM-affected rhesus macaques had at least one-half sibling diagnosed with HCM sharing 15 dams and 51 sires. Six HCM-affected rhesus macaques had full-siblings diagnosed with HCM. Of 143 rhesus macaques with HCM, the phenotype status of 23 parents were known. Ten additional parents were also screened with echocardiography, but they were excluded due to their equivocal HCM status. Eleven of the 23 rhesus macaques' parents with known phenotype status (47.8 %) were diagnosed with HCM. Of 502 rhesus macaques without HCM, 86 parents were screened for HCM status. There were 33 out of 86 (38.3 %) rhesus macaques with known HCM status were diagnosed with HCM that were parents of unaffected rhesus macaques. There were 22 sires having multiple offspring with HCM and there were 11 dams having multiple offspring with HCM. The mean and median heritability estimate with a 95% confidence interval (CI) for HCM in rhesus macaques was 0.5 and 0.51 (95% CI; 0.14–0.82), respectively.

**Figure 1:**
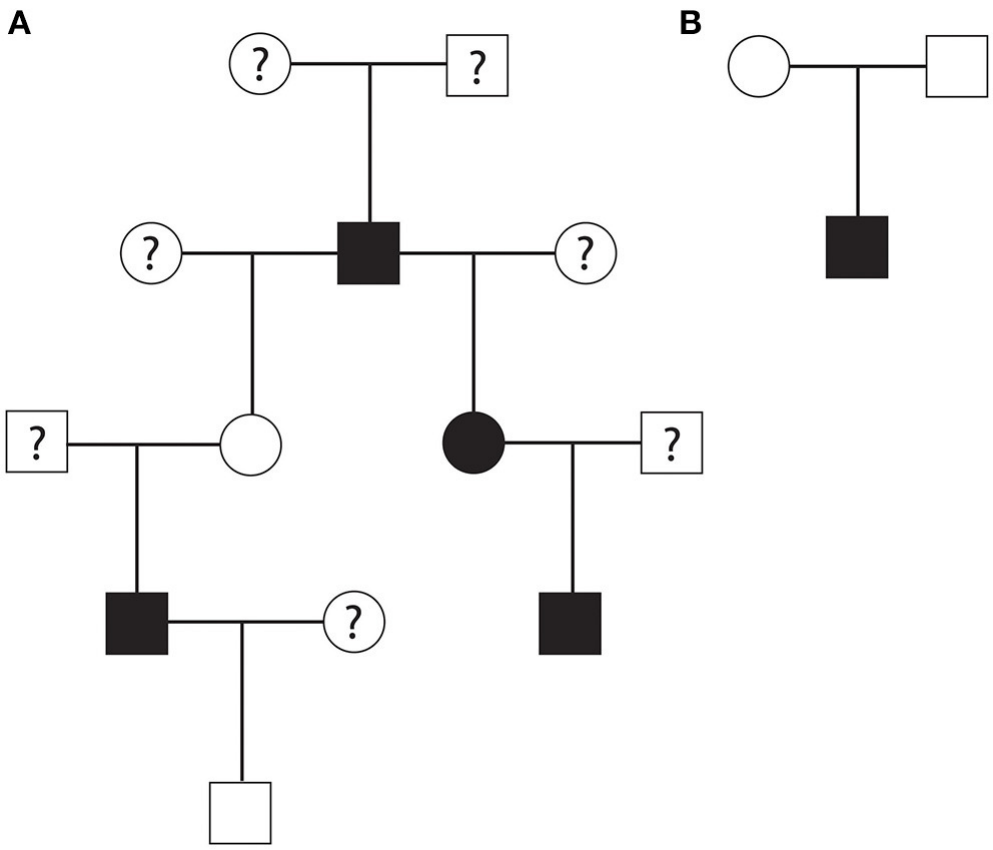
**(A)** A part of rhesus macaque family pedigree that suggested an autosomal recessive mode of inheritance or autosomal dominant mode with incomplete penetrance for HCM. Another nuclear family pedigree **(B)** showing that non-HCM affected parents produced one HCM-affected male rhesus offspring suggesting autosomal recessive mode of inheritance. Square; male, circle; female, white; no HCM, black; HCM, question mark; unknown HCM status.

A part of the overall pedigree is shown in Figure 1. The pedigree tree (Figure 1A) includes a family of rhesus macaques spanning four generations. In this family, an alpha male produced three HCM-affected offspring (one male and two females) and four non-HCM offspring (and three equivocal cases). Two offspring of this alpha male rhesus macaque produced two HCM-affected males. In another nuclear family, a non-HCM male and female produced HCM-affected male animal (Figure 1B). The mode of inheritance is less likely X-linked because of evenly distributed cases of HCM in both males and females. The pedigree analysis suggested a possible autosomal recessive mode of inheritance due to the fact that the nuclear family had an HCM-affected male from non-HCM parents. However, autosomal dominant mode of inheritance with incomplete penetrance is also possible.

## Discussion

The genetic contribution of developing HCM in rhesus macaques was characterized by performing a pedigree and heritability analyses in this study. The mean and median heritability estimate was 0.5 and 0.51, respectively, in this population with a 95% confidence interval of 0.14–0.82. This suggests genetics influence development of HCM in the colony of rhesus macaques at the CNPRC. However, post-translational modifications such as phosphorylation of mitogen-activated protein kinase, and environmental factors, such as diet and behavioral characteristics, are also likely to contribute the variability of phenotypes of this disease and may be responsible for the variability of phenotypic expression (25–28). Due to wide range of the 95% CI obtained in this study, additional screening of HCM in siblings, offspring, and parents of HCM-affected rhesus macaques is necessary to further determine the impact of genetic and environmental factors on development of HCM in rhesus macaques.

Heritability is a statistical measure of how much of variations in phenotypes can be attributed to variations in genotypes, and this is represented as a h^2^ value ranging from zero to one. The heritability of a phenotype is generally specific to a population investigated, and this estimation varies in different populations with different circumstances (29). In general, the higher a h^2^ value is, the higher the genetic influence on a trait. Many inherited diseases in humans due to mutations in a single gene have high heritability closer to one, but most complex traits in people have heritability somewhere in the middle around 0.5 indicating these disease variabilities is due to a combination of genetic and environmental factors (29, 30).

HCM in humans is known to be caused by mutations of various sarcomeric genes with autosomal dominant inheritance with incomplete penetrance (11). However, recent studies suggest that other subsets of HCM patients are associated with various mutations in non-sarcomeric genes and likely to be polygenic disease (31, 32). This trait could be associated with various environmental factors including certain lifestyle, diet, and behavioral characteristics (6, 33). The mode of inheritance for these patients often does not follow the typical autosomal dominant. HCM in humans is considered as a complex disease with incomplete penetrance or mixed mode of inheritance with various epigenetic and post-translational influences including DNA methylation, and acetylation of histones as well as phosphorylation and ubiquitination of signaling pathways. (25, 34–38). Therefore, the heritability, h^2^, is expected to be somewhere in the middle around 0.5 or less. In one study, a heritability estimation was performed in monozygotic twin pairs who share 100% of their genome and control pairs of same-sex dizygotic twins or same-sex normal siblings, who share 50% of their genomes. In that study, the heritability (h^2^) of HCM was 0.0% (95% CI 0.0–43.6%) (29). This finding suggests that differences in disease development and its severity in monozygotic twin pairs with a known mutation causing HCM is significantly influenced by post-translational modifications or environmental factors. It should however be noted that this study was performed based on IVSd associated with HCM, and the heritability estimation (h^2^) could be significantly altered if other traits were used to calculate h^2^. Heritability estimation was also performed for various cardiovascular diseases and was estimated around 0.2–0.4 for complex diseases such as systemic hypertension, dilated cardiomyopathy, and arrhythmias (12). To the author's knowledge, no studies have looked at actual heritability in a large population of HCM in human patients, but it could be similar to the finding in the present study. In this study, no variance component analysis was performed due to limited pedigree and environmental data to support this approach. Our previous studies to investigate the characterization of HCM in rhesus macaques did not reveal any significant variants within HCM phenotypes (16–19, 39). Nevertheless, future heritability and pedigree analyses in this colony should aim to include more phenotypic data of nuclear families, siblings, and offspring of rhesus macaques with HCM. Additionally, detailing shared and unshared environmental factors in a nuclear family, siblings, offspring, and across the entire colony could improve the accuracy of heritability analysis in a future study.

One alpha male rhesus macaque had three HCM-affected offspring and at least one HCM case was found in three sequential generations in this study. However, HCM status was not obtained from the majority of dams with HCM-affected offspring and therefore the mode of inheritance was not able to be determined based on the pedigree analysis of this family. There is a nuclear family which included HCM affected offspring with non-HCM parents. This suggests possible autosomal recessive mode of inheritance of HCM in rhesus macaques. However, autosomal dominant trait with incomplete penetrance cannot be ruled out without additional data of HCM-status on siblings, offspring, and parents of HCM-affected cases. In addition, HCM could develop with increased age of the rhesus macaque due to age-related penetrance, post-translational modifications, environmental factors, and patient behavioral factors, and thus the control rhesus macaques could develop HCM later in their life. Further study therefore should include longitudinal follow up study of HCM in this colony to determine the true prevalence of HCM and its heritability as well as the mode of inheritance.

There are a few limitations that need to be noted here. Heritability estimation is largely dependent on the sample size. Although more than 1,500 rhesus macaques were involved in this study, about a half of the individuals did not have any information about HCM status. By having more animals screened for HCM using echocardiography and other antemortem analyses, more accurate heritability estimation with a narrower 95% confidence interval could be obtained. In addition, the heritability estimate could vary in different subgroups of the population and this should be performed in younger and older rhesus macaques to eliminate the factor associated with the onset of age. Other variables, including environmental factors and physiological and psychological characteristics of animals, were also not added as covariates to the heritability and pedigree analyses due to the small sample size with known phenotypes in a nuclear family, siblings, and offspring of HCM macaques. Further characterization of potential individual and environmental factors would be required to determine the heritability with a narrower confidence interval in a future study. The mode of inheritance of HCM in rhesus macaques was not completely determined due to limited data obtained from full and half siblings, offspring, and parents of HCM cases. With additional dataset from these relatives of HCM-affected rhesus macaques, the mode of inheritance could be more accurately determined in the future. Nevertheless, it is possible that the HCM in rhesus macaques has the mixed mode of inheritance as in some subgroups of HCM in people.

In this study, the mean and median heritability (h^2^) was calculated as 0.5 and 0.51, respectively. This finding indicates that HCM in rhesus macaques is likely to be at least partially attributed by genetic variants with a significant environmental or post-translational influence on the development of HCM phenotypes in rhesus macaques at the CNPRC. The mode of inheritance was not completely determined based on the present study, but the pedigree analysis suggested an autosomal recessive mode of inheritance. However, autosomal dominant trait with incomplete penetrance cannot be ruled out with the data obtained. Further pedigree analysis should be performed with including more HCM and non-HCM cases for parents, siblings and offspring of HCM-affected cases. Since genetic influences on development of HCM in rhesus macaques was highly suggested based on the present study, further study should include findings of causative variants of HCM in rhesus macaques by performing whole genome or exome sequencing as well as performing an expression analysis to elucidate molecular pathways. The results of future studies will allow us to develop the first non-human primate model of naturally occurring HCM into a valuable research resource that will fuel discovery and intervention for HCM in human patients.

## Data Availability Statement

The datasets generated for this study are available on request to the corresponding author.

## Ethics Statement

The animal study was reviewed and approved by Institutional Animal Care and Use Committee of the University of California-Davis.

## Author Contributions

YU, JS, RR, and JR conceived the research. JS, RR, and YU selected the study subjects. YU and RR collected the data. YU and SK analyzed the data. YU wrote the manuscript. All authors contributed to the article and approved the submitted version.

## Conflict of Interest

The authors declare that the research was conducted in the absence of any commercial or financial relationships that could be construed as a potential conflict of interest.

## Abbreviations

CNPRC: california national primate research center
HCM: hypertrophic cardiomyopathy
I/O ratio: left ventricular internal to outer diameter ratio.
